# Structural and functional brain alterations in patients with hyperacusis: MRI systematic review

**DOI:** 10.3389/fnhum.2026.1785826

**Published:** 2026-04-01

**Authors:** Rania Alkahtani, Reem Elbeltagy, Zuhal Y. Hamd, A. B. Abdoelrahman Hassan

**Affiliations:** 1 Department of Health Communication Sciences, College of Health and Rehabilitation Sciences, Princess Nourah bint Abdulrahman University, Riyadh, Saudi Arabia; 2 Department of Radiological Sciences, College of Health and Rehabilitation Sciences, Princess Nourah bint Abdulrahman University, Riyadh, Saudi Arabia; 3 Department of Radiotherapy, College of Medical Radiologic Sciences, Sudan University of Science and Technology, Khartoum, Sudan

**Keywords:** DWI, hyperacusis, magnetic resonance imaging, neuroimaging, tinnitus

## Abstract

Hyperacusis is a condition marked by increased sensitivity to everyday sounds, often resulting in discomfort, pain, and social withdrawal. Despite its significant impact on mental well-being and quality of life, the underlying neural mechanisms remain poorly understood. This systematic review evaluates magnetic resonance imaging (MRI)-based studies investigating structural, functional, and connectivity-related brain changes in individuals with hyperacusis. A total of 11 studies were included, using structural MRI (sMRI), functional MRI (fMRI), and diffusion tensor imaging (DTI). Functional MRI studies consistently reported increased neural activity in key auditory regions such as Heschl’s gyrus, superior temporal gyrus, and the parahippocampal area. These findings were associated with high standardised mean differences (SMDs >5.0), reflecting exaggerated auditory processing. Structural MRI findings showed reduced grey matter volume, particularly in the right supplementary motor area, with large SMDs (e.g., SMD = 2.10), suggesting impaired sound modulation mechanisms. DTI studies highlighted altered integrity of auditory pathways, including the medial geniculate nucleus and inferior colliculus, supporting the presence of disrupted connectivity. Subgroup analyses revealed modality-specific effects, with sMRI emphasising cortical alterations and fMRI capturing network-level hyperactivity. High heterogeneity in pooled estimates was attributed to differences in imaging protocols, study designs, and analysis methods. Sensitivity analyses helped stabilise the findings by accounting for extreme values. The review concludes that hyperacusis is a complex, multisystem condition involving both auditory and emotional processing networks. These findings underline the importance of adopting integrated diagnostic protocols and multidisciplinary therapeutic strategies targeting neural, emotional, and perceptual dysfunctions.

## 1 Introduction

Hyperacusis is an auditory disorder in which the patient develops a high sensitivity toward sound, which, in most cases, results in discomfort, pain, and anxiety. Clinically, hyperacusis is categorised into subtypes based on the dominant symptom: loudness hyperacusis (increased perception of volume), pain hyperacusis (physical pain from sound), annoyance hyperacusis (emotional irritation), and fear hyperacusis (sound-induced anxiety or phobia). These subtypes often overlap and contribute to the heterogeneity of patient experiences. Recognising these distinctions is essential for accurate diagnosis and targeted intervention (Baguley et al., 2014). Unlike other auditory disorders, such as tinnitus, hyperacusis authenticity towards sounds increased to intolerable levels, disrupting daily activities and affecting the quality of life (Sheppard et al., 2020). High sensitivity towards sound severely affects Social and professional interactions, as the patient suffering from hyperacusis tries to avoid social gatherings and crowded places (Makani et al., 2023). Around 16 million to 1.3 billion people suffer from this condition worldwide (Ren et al., 2021); this auditory disorder affects the whole life of the person. Still, the underlying mechanism of hyperacusis is unknown; this disorder is not limited to peripheral auditory anomalies but involves complex central auditory processing (Tyler et al., 2014), which is unknown.

Several neuroimaging studies using magnetic resonance imaging (MRI) have provided significant insight into the neural basis of hyperacusis. sMRI studies indicated a reduction in grey matter volumes in key auditory regions of the superior temporal means, which indicates the structural changes associated with sound processing abnormalities (Salvi et al., 2022). Changes observed in the insula and anterior cingulate cortex are known to play a role in emotional responses, which suggests hyperacusis may also connected to emotional processing networks (Schecklmann et al., 2014). Hyperacusis might be a multisystem illness involving auditory and emotional brain networks (Eggermont, 2021).

Aberrant brain activity patterns in hyperacusis patients are additionally understood by functional magnetic resonance imaging (fMRI) investigations. Studies find that the brain’s auditory cortex, thalamus, and amygdala regions are activated during sound exposure, suggesting increased auditory processing and stress reaction (Makani et al., 2024). Altered connectivity in networks such as default mode networks and silence networks highlighted potential dysfunctions in sensory integration and attentional regulation, which are necessary to handle sensory inputs (Jimoh et al., 2023). These studies suggest that sensory integration involving neural circuits that extend beyond the auditory system is disrupted by hyperacusis (Shahsavarani et al., 2019).

The integrity of white matter in individuals, which plays a vital role in auditory information studied by DTI, could provide additional insight into hyperacusis. White matter changes specifically, which play essential roles in auditory information transmissions like the arcuate fasciculus and corpus callosum, are identified as significant pathways (Tai et al., 2023; Kim et al., 2023). Neural disruption contributes to complex clinical presentation linked with hyperacusis and is supported by these alterations in neural pathways (Tyler et al., 2014). As auditory, emotional, and cognitive processes are also affected by hyperacusis, it is mainly recognized as multifaceted disorders that require systematic evaluation and intervention strategies (Koops and van Dijk, 2021).

In this systematic analysis, we aim to summarize the results of all MRI-based research focused on hyperacusis, emphasizing changes in the brain’s structure, function, and connection. This review can provide an understanding of the underlying mechanism of hyperacusis, enhancing the diagnostic accuracy and development of targeted interventions such as cognitive-behavioral therapy, neural biomarkers, and pharmacological treatments. This data might help address various difficulties faced by hyperacusis patients, and this could also be used to build a personalized and deeper comprehensive brain analysis that can provide effective treatment strategies.

## 2 Methods

The systematic literature review used the PRISMA guidelines (Moher et al., 2015) and PICO statements to identify, select, and analyze the studies on brain changes in hyperacusis patients using MRI. Meta-analysis was conducted to find the quantitative MRI-based studies on brain changes in hyperacusis. The following is the definition of the PICO criteria: Adults with hyperacusis were included in the population (P); for intervention(I), different MRI modalities (such as sMRI, fMRI, and DTI) were chosen; participants with other auditory conditions or healthy control subjects (C) served as comparators; and for the outcome (O) main focus was on change in brain structure, function, and connectivity.

### 2.1 Literature survey strategy

We systematically searched the PubMed and Scopus databases for all the studies investigating brain changes using MRI in patients with hyperacusis. This review included all the literature published from May 2002 to 9th October 2024. We used the combination of Medical subject headings (MeSH) terms and keywords to find relevant studies. The following terms were used for the search strategy- Hyperacusis, Sound sensitivity, Auditory hypersensitivity, Magnetic Resonance Imaging, MRI, fMRI, DTI, Structural MRI, Brain structure, Brain volume, Cortical thickness, Functional connectivity, and White matter integrity. Both controlled vocabulary of MeSH and accessible terms were employed to ensure the comprehensive findings of relevant studies (Cumpston et al., 2019; Bramer et al., 2017; Yong et al., 2017).

### 2.2 Selection criteria

#### 2.2.1 Inclusion criteria

Selection criteria for inclusion included peer-reviewed articles that used MRI neuroimaging to analyze brain changes in patients diagnosed with hyperacusis. Selected studies must have adult participants (18 years or older) and should have a well-defined control group of healthy individuals for comparison. Furthermore, all studies should have quantitative metrics for MRI containing data for brain volume, cortical thickness, or functional connectivity. Studies published only in English and peer-reviewed journals were included to maintain the quality of the meta-analysis (Muka et al., 2020; Schizophrenia Research, n.d.; McClung Pasqualino, 2020).

#### 2.2.2 Exclusion criteria

For the exclusion criteria, studies in which pediatric patients, lack of MRI use, control group were missing, qualitative-only assessments, other primary auditory disorders (e.g., tinnitus without hyperacusis), non-peer-reviewed articles, and studies published in other than English language were selected. Due to overlapping characteristics, the scope was broadened to include relevant hyperacusis studies with tinnitus and the search strategy was adjusted accordingly to reflect this inclusion (Gogtay and Thompson, 2010; Whitfield-Gabrieli and Ford, 2012).

### 2.3 Data extraction

Data were extracted with information on MRI techniques (scanner type, analysis method, imaging sequences), sample size, population characteristics (hyperacusis severity, age, gender, comorbidities), study design (cross-sectional, cohort), outcomes (primary and secondary), study title, and year. All of this information was collected carefully. Key findings of changes in brain regions and statistical results were noted for the review. To ensure consistency, data extraction was carried out by two independent researchers using a standardized form. Disparities were sorted through discussions to settle disagreements. The extraction form was pilot-tested to provide a comprehensive data collection with precision and clarity, allowing for the thorough gathering and analysis of pertinent data (Higgins et al., 2011b).

### 2.4 Risk of bias statement

**Figure 1:**
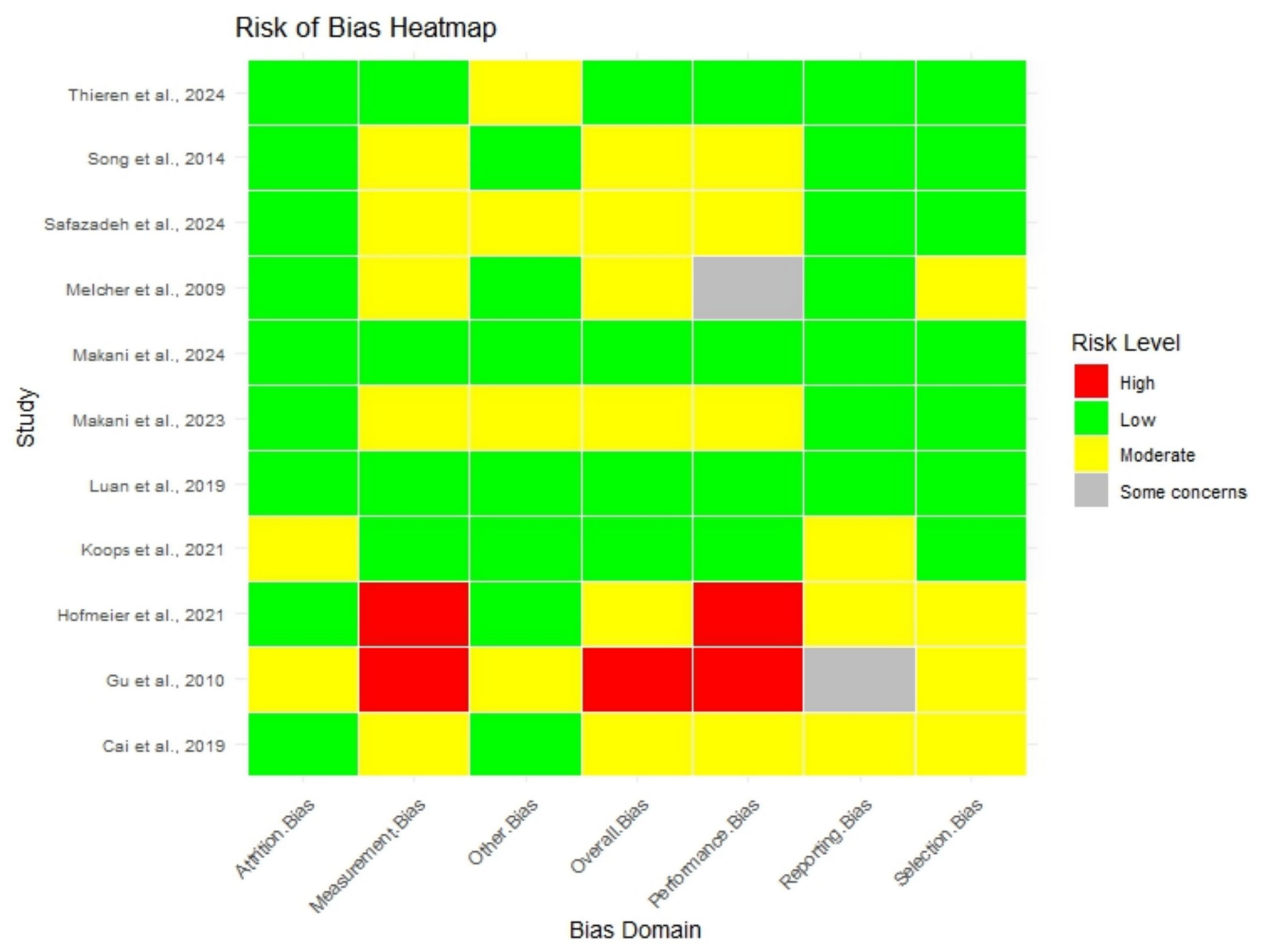
ROB Analysis of selected studies.

For each study that the authors deemed appropriate, the risk of bias was evaluated systematically across six domains in this review: Selection Bias, Performance Bias, Detection Bias, Attrition Bias, Reporting Bias, and Other Biases. Each domain was assessed concerning pre-established criteria, the levels of risk being classified as Low, Some Concerns, Moderate, or High. Selection Bias was assessed regarding the recruiting strategies to reduce any selection risk. In considering Performance Bias evaluation, careful attention was paid to the precise description of study interventions, implementation of the interventions, and adherence to study protocol. In assessing the risk of Detection Bias, the review determined how outcome measurements would be performed and how the outcomes would be quantified to achieve objective outcomes assessment. As for Attrition Bias, the completeness of data was addressed by considering participants’ losses or withdrawals during the research. Reporting Bias concerns the quality, completeness and currency of outcomes reported. Other Biases were factors that may distort study results and are not linked to the study design, such as confounding factors. The risk of bias was rated by various authors independently. Any differences between authors were resolved after discourse to achieve agreement. This systematic method enabled a uniform approach while evaluating the included studies despite the different methods adopted by the studies per the regulations (Higgins et al., 2011a; Sanderson et al., 2007; Verhagen et al., 1998; Savović et al., 2014), ROB Analysis of selected studies demonstrated in Figure 1. Using Heatmap for risk assessment.

### 2.5 Prisma plot

PRISMA plot 2020 flow diagram was used to document the literature selection. Databases searched, such as PubMed and Scopus, were used to find studies. After removing 22 duplicates, 116 records were recorded and screened for title and abstract for review. In the first screening, based on abstract and title, 46 articles were removed. All of the remaining 70 studies were retrieved successfully. Full-text was evaluated, 59 were removed, and 11 were selected as in Table 1, by the following criteria: absence of MRI data (*n* = 9), a lack of a control group (*n* = 17), no human (*n* = 2), and studies without hyperacusis (*n* = 21) (Liberati et al., 2009).

**Table 1:** Literature table showing the details of selected studies.

Study	Population	Intervention	Comparison	Outcome	Sample size
Safazadeh et al. (2024)	18 Tinnitus (normal hearing), 20 Controls	fMRI with monaural pure tone stimuli (353–8,000 Hz)	Tinnitus patients vs. healthy controls	Increased lateral auditory cortex activity; hyperactivity at low frequencies; tinnitus interaction in parahippocampus	38
Makani et al. (2024)	101 tinnitus (35 normal hearing, 66 hearing loss); hyperacusis subgroup analysis	Structural MRI (gray matter volumes)	Tinnitus with hyperacusis vs. tinnitus without hyperacusis	Smaller SMA gray matter volumes associated with hyperacusis	101
Melcher et al. (2013)	32 volunteers (12 tinnitus, 20 non-tinnitus)	fMRI with broadband noise stimuli	Tinnitus subjects vs. matched controls	Increased inferior colliculus activation in tinnitus group indicating abnormal auditory pathway gain	32
Makani et al. (2023)	73 tinnitus with sensorineural hearing loss (SNHL)	Structural MRI (gray matter morphology)	Tinnitus patients with vs. without hyperacusis	Reduced gray matter volumes and cortical thickness in right SMA; increased sound-evoked responses in SMA with hyperacusis	73
Koops (2021)	35 tinnitus with hearing loss (with and without hyperacusis)	fMRI and loudness matching tasks	Hearing loss with hyperacusis vs. hearing loss without hyperacusis	Higher subcortical and cortical auditory activity; reduced response at tinnitus frequency in hyperacusis group	35
Gu et al. (2010)	27 subjects (13 tinnitus, 14 controls)	fMRI and behavioral sound-level tolerance testing	Hyperacusis vs. subjects with normal tolerance	Elevated auditory midbrain, thalamus, and primary auditory cortex activation linked to hyperacusis; auditory cortex activation related to tinnitus	27
Song et al. (2014)	34 tinnitus (17 hyperacusis, 17 non-hyperacusis), 17 healthy controls	Resting-state EEG (electroencephalography)	Tinnitus patients with hyperacusis vs. tinnitus without hyperacusis	Increased beta power in dACC and OFC; increased alpha power in auditory cortex; altered functional connectivity in hyperacusis	51
Thieren et al. (2024)	30 hyperacusis (15 men, 15 women)	Cognitive Sound Exposure Therapy (CSET)	Before vs. after therapy	Reduced hyperacusis symptoms, improved sound tolerance and decreased sensitivity to everyday sounds	30
Cai et al. (2019)	31 tinnitus (16 normal hearing, 15 healthy controls)	Resting-state fMRI (smALFF)	Tinnitus patients with normal hearing vs. healthy controls	Increased spontaneous activity in auditory cortex, altered connectivity with limbic and motor regions	31
Luan et al. (2019)	35 SNHL vs. 35 healthy controls	fMRI and DTI (Diffusion Tensor Imaging)	SNHL vs. healthy controls	Disrupted functional connectivity and decreased white matter integrity in auditory and frontal-temporal pathways	70
Hofmeier et al. (2021)	50 tinnitus patients (20 with hyperacusis) and 43 controls	ABR (Auditory Brainstem Response) and BOLD fMRI	Tinnitus patients with vs. without hyperacusis	Higher ABR wave III and V amplitudes in hyperacusis; increased BOLD fMRI responses in MGB, AC-I, and sound identification regions	93

### 2.6 Forest plot and meta-analysis

The analysis included studies of MRI outcomes, which can be compared quantitatively with brain volume, cortical thickness, or functional connectivity. Depending on the measured outcomes, the effect sizes were extracted from each study, including mean differences, odds ratios, or coefficients.

#### 2.6.1 Data synthesis statistical analysis

Meta-analysis was conducted by using a random-effects model for accounting variability across studies. The outcome of primary measures was pooled using assessing effect sizes (Cohen’s d) and 95% confidence intervals (CIs). *I*^2^ statistics, with 25, 50, and 75% representing low, moderate, and high heterogeneity, respectively, were measured to assess heterogeneity. For Cochran’s Q-test, a *p*-value <0.10 was regarded as suggestive of considerable heterogeneity (Cochran’s Q-test, n.d.; Higgins and Thompson, 2002).

#### 2.6.2 Forest plot construction

Forest plots were generated using R with the effect sizes from individual studies and overall pooled effects for visual representation. All plots display the estimation of effect size (Cohen’s d), 95% CI, and the weight assigned to each study within the analysis. Studies were grouped according to the results, for example, grey matter volume or functional connectivity, then stratified by MRI technique (sMRI, fMRI, DTI, etc) (Borenstein et al., 2009; Altman, 1991; Greenland, 1987). The formula for Cohen’s *d* is:


$$d=\frac{M_{1}-M_{2}}{SD_{\text{pooled}}}$$

*SD_pooled_* is the pooled standard deviation, calculated as:


$$SD_{\text{pooled}}=\sqrt{\frac{(n_{1}-1)\cdot SD_{1}^{2}+(n_{2}-1)\cdot SD_{2}^{2}}{n_{1}+n_{2}-2}}$$

Where *M*_1_ = Mean of the patient group, *M*_2_ = Mean of the control group, *SD*_1_ = Standard deviation of the patient group, *SD*_2_ = Standard deviation of the control group, *n*_1_ = Number of patients (sample size of the patient group), n_2_ = Number of controls (sample size of the control group).

### 2.7 Software

All the statistical analyses for the meta-analysis, forest plot generation, and publication bias assessments were conducted using Python and R. After analysis, the PRISMA plot was developed using.^1^ This website and forest plot were generated using Python.

## 3 Results

### 3.1 Study selection

Studies for systematic review were selected by following PRISMA guidelines and presented using a PRISMA flow diagram. From 138 searched articles, 11 studies met all the criteria of adult participants, a well-defined healthy control group, MRI studies with details, and peer-reviewed journals published in English.

### 3.2 Study characteristics

Selected studies addressed a broad range of hyperacusis characteristics. These studies selected patients who are primarily adults with a mix of hearing loss and healthy control. These research articles evaluated various outcomes, including grey matter changes, connectivity patterns, and functional activation within the central auditory system and related regions. fMRI, sMRI, DTI, and EEG were employed to capture the neural correlates with hyperacusis (Rauschecker et al., 2010; Husain and Schmidt, 2014; Carpenter-Thompson et al., 2015; Joos et al., 2012; Safazadeh et al., 2024).

### 3.3 MRI findings

The consolidated findings of MRI from 11 studies represent a systematic and connected view of the functional and structural changes linked with hyperacusis. These studies highlight complex neural mechanisms underlying the hyperacusis characterized by central auditory gain and altered connectivity.

#### 3.3.1 Structural MRI

sMRI results consistently suggested a significant reduction in grey matter volume in critical areas of auditory processing. Studies have also highlighted the anatomical deficiencies in the brainstem and central auditory pathways and discovered that central demyelination in multiple sclerosis patients contributes to hyperacusis, highlighting the structural deficits in the brainstem and central auditory pathways (Scheerer et al., 2022). These findings were consistent with the other publications where it was reported that the decrease in areas of grey matter, such as the insula, orbitofrontal cortex, and medial geniculate nucleus, is essential to regulating senses and emotions (Mahoney et al., 2011; Boucher et al., 2015). Furthermore, reduced grey matter in the right supplementary motor area (SMA) highlights that motor areas play a role in modulating auditory responses modulation (Makani et al., 2023). The studies found apparent declines in grey matter volume and cortical thickness in the important areas for hearing and emotional processing in patients with hyperacusis. In particular, the right supplementary motor area (SMA) had decreased grey matter volumes, which can associate changes in structure with hearing problems in hyperacusis patients.

#### 3.3.2 Functional MRI

In the medical geniculate body and primary auditory cortex, excessive neural activation was observed in fMRI front studies (Boyen et al., 2013). Central auditory gain, which is the excessively amplified sound signals along the auditory pathways, can be concluded from this finding. Increased neural activity was not only restricted to cortical regions; similar hyperactivity was also observed in inferior colliculus and wider cortical networks (Koops and van Dijk, 2021; Gu et al., 2010). This highlighted that sound frequency is not influenced by more widespread sensory impairment. Functional magnetic resonance imaging (fMRI) research has also identified abnormally activated neural activity patterns in hyperacusis patients (Thieren et al., 2024). Gu et al. (2010) and Hofmeier et al. (2021) showed increased activation of the auditory midbrain, thalamus, and primary auditory cortex (PAC) in response to acoustic stimulation (Gu et al., 2010; Hofmeier et al., 2021). Song et al. (2014) also found increased beta power in the dorsal anterior cingulate cortex (dACC) and orbitofrontal cortex (OFC) that correlated with the severity of hyperacusis and indicated involvement of these regions in increased sound input sensitivity (Song et al., 2014). These findings are further supported by Cai et al. (2019) who also found an increase in spontaneous neural activity (smALFF) in the higher auditory cortex (HAC) and altered connectivity between auditory networks and emotion regulation networks, including the hippocampus and amygdala (Cai et al., 2019).

#### 3.3.3 Diffusion tensor imaging

Understanding the connectivity disruption of key auditory pathways by DTI can give a better overview of the hyperacusis mechanism. Variations in fiber tract integrity were mainly associated with pain-related hyperacusis, suggesting disruptions in neural pathways connected to pain processing. Altered semicircular canal fluid signals in semicircular canal (SSCD) patients are linked with changes in hyperacusis (Carpenter-Thompson et al., 2015). These findings suggested alterations in hyperacusis symptoms, indicating that auditory pathways’ basic structure may be changed. Hyperacusis neurological underpinning is a complicated scenario as this abnormality in fiber tract integrity is pain-related; this suggests that hyperacusis involves both hypersensitivity and broader sensory integration issues, emphasizing the need for multisystem approaches for diagnosis and treatment (De Ridder et al., 2011; Brantberg and Löfqvist, 2007).

Overall, the structural and fMRI results highlight hyperacusis as a disorder marked by a condition in which extensive activation, excessive neural gain, and altered connections between auditory and non-auditory networks were observed. The insights highlighted the connection between anatomical deficiencies and more excellent neural responses, indicating that hyperacusis is driven by the altered brain circuits regulating signals and the improved processing of sensory inputs. The evidence supports that a comprehensive model of hyperacusis needs a multi-approach approach for therapeutics (De Ridder et al., 2011; Brantberg and Löfqvist, 2007). Luan et al. (2019) revealed significant disruptions in white matter integrity within key auditory pathways, such as the inferior frontal-occipital fasciculus (IFOF), inferior longitudinal fasciculus (ILF), and superior longitudinal fasciculus (SLF) (Luan et al., 2019). These alterations indicate compromised neural pathways potentially underlying hyperacusis symptomatology and contributing to broader sensory integration deficits.

### 3.4 Forest plot and meta-analysis

**Figure 2:**
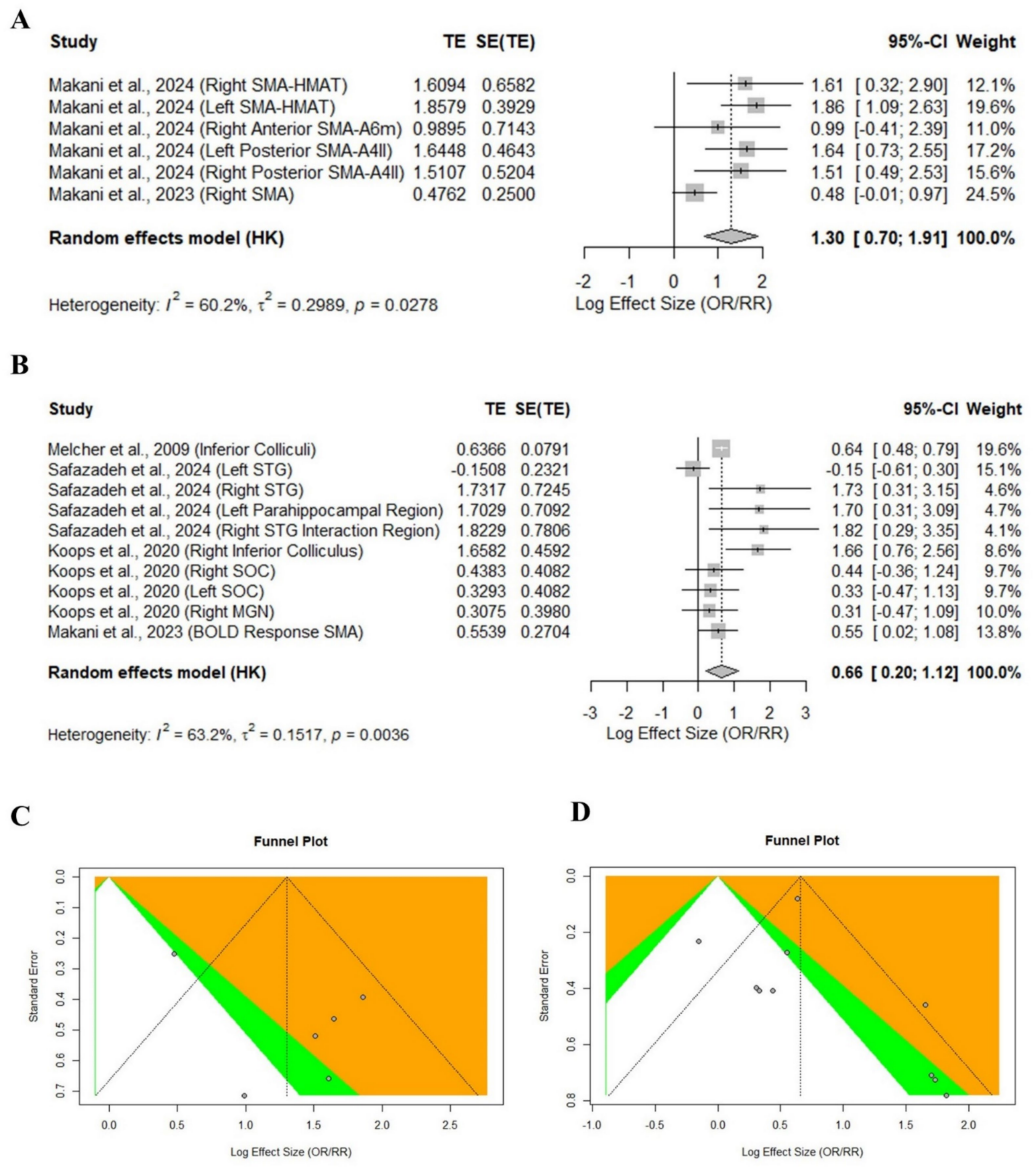
Meta-analysis of selected studies with available numerical values: **(A)** Forest plot of Gray matter volume; **(B)** Forest plot of Sound evoked Activation; **(C,D)** Their respective Funnel plots.

Structural and fMRI observations on brain changes in hyperacusis patients are summarized using the forest plot for multiple studies. In Figure 2A Significant changes in different regions of the brain that are responsible for auditory responses and emotional processing were noted. fMRI studies revealed that increased sensitivity in several key auditory areas of the superior temporal gyrus, the left parahippocampal region, the left Heschl’s gyrus, and the right superior temporal gyrus exceeded a high SMD of 5.0. These findings highlight that in hyperacusis patients, the activation of auditory processing critical regions was observed (Adams et al., 2020; Vermeulen, 2018). Furthermore, differences in the inferior colliculus, superior olivary complex (SOC), and medial geniculate nucleus (MGN) emphasize the central auditory pathway disruptions. The pooled SMD of auditory response-related areas was 0.66 [0.20, 1.12], with a moderate effect size, and heterogeneity (*I*^2^ = 63.2%), showing variability between studies (Figure 2A).

Figure 2B suggests that sMRI findings indicate a reduction in grey matter volume and cortical thickness, specifically in the right supplementary motor area. This suggests the alterations in structure may influence sound modulation. Additionally, sMRI and DTI observations suggested the differences in the superior olivary complex, medial geniculate nucleus, and right inferior colliculus, emphasizing the disruption in auditory pathways in hyperacusis patients. These findings suggest structural alterations potentially influencing sound modulation in patients with hyperacusis. Notably, a large SMD of 1.86 [1.09, 2.63] was observed in the left SMA-HMAT, and the right SMA-HMAT and posterior SMA regions also had large reductions. The overall SMD for gray matter alterations was 1.30 [0.70, 1.91] with heterogeneity (*I*^2^ = 60.2%), describing moderate study heterogeneity (Chen et al., 2015; Lin et al., 2020). Figures 2C,D show the funnel plots used to assess potential publication bias across the included studies.

Initially, analyzing all studies together yielded extremely high heterogeneity (*I*^2^ = 96.5%), suggesting substantial variability across methods and populations. By subgrouping according to anatomical categories, we observed a marked reduction in variability for certain regions. When studies focused on primary auditory structures, the heterogeneity dropped to 0%, and a similar reduction to *I*^2^ = 0% was seen in secondary auditory/association cortices. This dramatic decrease implies that mixing diverse brain regions drives much of the initial variability. Although the motor and supplementary motor areas remained heterogeneous, the results demonstrate that anatomically informed subgrouping can isolate more uniform findings. Consequently, the pooled estimates in these anatomically defined subgroups became more stable, interpretable, and reliable, underscoring the importance of careful subgroup selection in neuroimaging meta-analyses. As supported by findings, hyperacusis might involve multiple pathways of structural and functional alterations in auditory and emotional regulation and requires a multidisciplinary approach that targets both auditory and emotional regulatory pathways for the diagnosis and therapeutics (Lee et al., 2020; Eggermont and Roberts, 2015; Koops, 2021; Hofmeier, 2021).

## 4 Discussion

In this study, we have reviewed the evidence of structural and functional brain changes amongst hyperacusis patients, which further provides an understanding of the neural conduits of this auditory sensitivity disorder (Husain and Schmidt, 2014; Boyen et al., 2013). The results that arise from different MRI modalities, which include sMRI, fMRI and DTI, report certain anomalies within specific brain regions that deal with sound processing and emotional regulation. The reduced volume of grey matter in areas such as the superior temporal gyrus, insula and orbitofrontal cortex suggests some structural impairment that enhances hearing ability (Gu et al., 2010). fMRI studies also show excessive activation of the primary auditory cortex, thalamus, and amygdala among hyperacusis sufferers, implying that sound processing and emotional response are overactive (Melcher et al., 2013).

Patients with hyperacusis exhibit distinct neural changes compared to healthy individuals and those with tinnitus. Structural imaging shows reduced grey matter in regions like the insula, superior temporal gyrus, and orbitofrontal cortex. Functional studies reveal increased activation in the auditory cortex, amygdala, and thalamus, indicating heightened central auditory gain and emotional response. Disrupted connectivity in the default mode and salience networks further reflects impaired sensory and attentional regulation. While tinnitus involves auditory pathway alterations (Möhrle et al., 2019), hyperacusis more prominently engages emotional circuits. These differences highlight the need for diagnostic and therapeutic strategies targeting both auditory processing and emotional regulation in hyperacusis (Husain and Schmidt, 2014; Leaver et al., 2016). This abnormality of connectivity may help to understand the reason for hyperacute sound perception, and hyper-emotional reactions in hyperacusis, which impairs the quality of life of patients (Carpenter-Thompson et al., 2015). Furthermore, some disruption in white matter structures, such as the arcuate fasciculus and corpus callosum, which are crucial to transmitting auditory information, is also depicted in the DTI studies, which infers that hyperacusis is a condition of hypersensitivity to sound and other forms of integration (Crippa et al., 2010).

### 4.1 Potential mechanisms underlying brain changes

Hyperacusis likely results from increased central auditory gain, where sound signals are excessively amplified, causing overactivation of auditory regions even at low volumes. This neural mechanism may explain why patients perceive normal sounds as intolerably loud, highlighting the role of dysfunctional auditory processing in the condition (Gu et al., 2010; Schaette and McAlpine, 2011; Knudson et al., 2014). One more mechanism could be the involvement of pathways in which both sensory and emotional networks interplay. The amygdala, a region responsible for emotional processing, might get highly active in hyperacusis patients, which suggests the brain can perceive harmless noises as threats (Rauschecker and Leaver, 2018; Jastreboff and Hazell, 1993). This might result in a “feedback loop,” in which the brain becomes more sensitive to sound as a result of emotional reactions being heightened by the perception of sound as threatening. This feedback loop can explain why hyperacusis patients frequently experience social withdrawal, and reduce quality of life and anxiety (Husain and Schmidt, 2014; Leaver et al., 2016; Fackrell et al., 2015). White matter connectivity disruptions, particularly in association fibers such as the arcuate fasciculus, SLF, and ILF, play a key role in hyperacusis pathophysiology. These tracts support integration between auditory, frontal, and language-processing regions. Altered integrity in these fibers may hinder top-down regulation of auditory input and contribute to exaggerated sound perception. Their involvement in speech and auditory attention also suggests a neural basis for the communication difficulties reported by hyperacusis patients. Degradation of association fibers, particularly those linking auditory and frontal regions, may impair top-down modulation of auditory input. This weakened control can lead to reduced filtering of external sounds, resulting in hypersensitivity. Given these fibers also support language and attentional processes, their disruption may contribute to the speech perception and communication difficulties commonly reported in hyperacusis. The integration of existing studies lends support to a more advanced neural structure of hyperacusis, with decreased structural integrity and increased functional responsiveness in the central auditory and limbic systems. Research by Hofmeier et al. (2021) provides robust evidence to infer that structural abnormalities, specifically in regions associated with auditory modulation (e.g., SMA, MGB, IC), may be responsible for a hyperacusis exaggeration of sound hypersensitivity (Hofmeier et al., 2021). Functional evidences indicate increased neural activity in auditory and limbic regions, which points towards hyperacusis as a complex condition that engages both auditory processing and emotional regulation networks (Gu et al., 2010; Hofmeier et al., 2021; Song et al., 2014; Cai et al., 2019).

### 4.2 Clinical implications

This review has key clinical implications. MRI findings support classifying hyperacusis as a multisystem disorder involving both sensory and emotional networks. Reduced grey matter in regulatory regions and altered connectivity patterns may help distinguish hyperacusis from other auditory disorders like tinnitus. These neuroimaging markers can aid in diagnosis and guide more targeted therapeutic strategies (Carpenter-Thompson et al., 2015; De Ridder et al., 2015).

Hyperacusis is associated with emotional and cognitive components, too, therefore, therapies targeting only auditory pathways will be insufficient for the treatment (Aazh and Moore, 2017). Therapies used for anxiety can help hyperacusis patients refrain from their emotional responses to sounds. Neuromodulation techniques might reduce the activation in the auditory cortex and lower sensitivity. Furthermore, white matter integrity auditory pathways could restore the auditory disorder. This approach to diagnosis and therapeutics will be more effective for hyperacusis (Knudson et al., 2014).

Given these findings, hyperacusis emerges clearly as a multisystem condition requiring comprehensive diagnostic and therapeutic approaches. Therapies exclusively targeting auditory pathways may prove insufficient; instead, interventions should incorporate strategies aimed at modulating limbic and attentional networks to effectively manage the emotional and cognitive dimensions of hyperacusis. The involvement of neural circuits identified by Song et al. (2014) and Cai et al. (2019), such as the OFC and dACC, also supports the potential utility of cognitive-behavioral therapy and neuromodulation techniques in addressing broader neural dysfunctions beyond auditory hyperresponsiveness alone (Song et al., 2014; Cai et al., 2019).

### 4.3 Broader implications

The overall interference of these observations makes our knowledge of sensory and auditory processing disorders more acceptable. Hyperacusis is similar to other auditory disorders and conditions characterized by increased sensitivity towards sounds, such as chronic pain, tinnitus, and some specific types of anxiety disorders (De Ridder et al., 2011; Folmer et al., 2015). These conditions cause similar symptoms in patients that involve hypersensitivity in neurons related to auditory processing, which suggests that they involve the common underlying mechanisms that could direct cross-disciplinary research on potential therapies. Immediately, techniques that have facilitated reduced auditory cortex activity in tinnitus, like transcranial magnetic stimulation (TMS) (Folmer et al., 2015), may also make investigating the hyperacusis worthwhile (Tyler et al., 2014; Jastreboff and Jastreboff, 2014). This systematic review emphasises the need for a paradigm shift in the hyperacusis way and similar conditions are viewed. It is not only an issue related to auditory dysfunction but is a complex, and multisystem disorder that involves the interplay of emotional, sensory, and cognitive pathways (Leaver et al., 2016; Knipper et al., 2013).

Studies using diffusion tensor imaging (DTI), such as done by Zolkefley et al. (2024), demonstrate that auditory-linguistic processing and multisensory integration may be significantly impacted by diminished integrity in long association tracts like the arcuate fasciculus (AF), inferior longitudinal fasciculus (ILF), and superior longitudinal fasciculus (SLF). Part of the dorsal language network, the AF facilitates speech production, phonological working memory, and repetition by connecting frontal speech-motor areas to posterior temporal auditory regions. Therefore, disruption of AF fibres may diminish the top-down regulation of auditory information, affect the ability to perceive speech in noise, and be a contributing factor to the communication problems that hyperacusis patients describe (Zolkefley et al., 2024)

## 5 Limitations

This review highlights several limitations. Most included studies were cross-sectional, precluding causal inferences. Sample sizes were generally small, limiting statistical power and generalisability. Patient heterogeneity (e.g., comorbid tinnitus, age, gender) adds variability. Substantial methodological differences, including variations in MRI protocols, scanner types, and analysis pipelines, particularly in DTI (e.g., tract-based vs. voxel-based approaches), limit comparability. The inclusion of studies with comorbid tinnitus may have influenced results. Due to inconsistent reporting, sensitivity analyses specifically excluding tinnitus cases were not possible, limiting hyperacusis-specific inferences. Medication use, such as SSRIs, was often unreported and may confound neuroimaging outcomes. Furthermore, potential publication bias may have t studies reporting significant findings, leading to an overrepresentation of positive results. These issues underscore the need for larger, standardised, and methodologically rigorous studies in hyperacusis research.

## 6 Conclusion

This systematic review synthesises current neuroimaging evidence on brain changes in hyperacusis, revealing structural and functional abnormalities across auditory and emotional pathways. Increased neural sensitivity, reduced grey matter in auditory regulatory areas, and altered white matter integrity support the role of abnormal auditory gain and disrupted connectivity. These findings reinforce the view of hyperacusis as a complex, multisystem disorder involving both sensory and non-sensory brain regions. Understanding these alterations can guide more accurate diagnosis and inform targeted interventions.

### 6.1 Future directions

Despite its clinical impact, the neurobiological basis of hyperacusis remains poorly understood. Future research should prioritise large-scale, longitudinal, and multimodal studies to determine causality and disease progression. Combining MRI with behavioural measures—such as speech-in-noise testing—may enhance diagnostic specificity by linking structural and functional alterations with real-world auditory performance. Further investigation into therapeutic interventions, including cognitive-behavioural and pharmacological strategies, is essential to assess their effect on brain function and reversibility. Integrating MRI with electrophysiological techniques or other imaging modalities may provide a more comprehensive understanding of hyperacusis subtypes and support the development of personalised treatment approaches.

## Data Availability

The original contributions presented in the study are included in the article/supplementary material, further inquiries can be directed to the corresponding author.

## Footnotes

1
https://www.prisma-statement.org/prisma-2020-flow-diagram
